# The use of healthcare systems data for RCTs

**DOI:** 10.1186/s13063-023-07846-4

**Published:** 2024-01-29

**Authors:** Alice-Maria Toader, Carrol L. Gamble, Susanna Dodd, Paula R. Williamson

**Affiliations:** 1 MRC-NIHR Trials Methodology Research Partnership, Department of Health Data Science, University of Liverpool, Liverpool, UK; 2 Liverpool Clinical Trials Centre, University of Liverpool, Liverpool, UK

**Keywords:** Healthcare systems data, Outcomes, Clinical trials, Routinely collected data, Data validity, Registries, Routinely collected health data

## Abstract

**Background:**

Healthcare systems data (HSD) has the potential to optimise the efficiency of randomised controlled trials (RCTs), by decreasing trial-specific data demands. Therefore, the use of HSD in trials is expected to increase. In 2019, it was estimated that 47% of NIHR-funded trials were planning to use HSD. We aim to understand the extent and nature of its current use and its evolution over time.

**Methods:**

We identified a cohort of RCTs within the NIHR Journals Library that commenced after 2019 and were described as being in progress on 6 June 2022. Details on the source and use of HSD were extracted from eligible RCTs. The use of HSD was categorised according to whether it was used as the sole data source for outcomes and whether the outcomes were primary or secondary. HSD is often insufficient for patient-reported outcomes (PROs). We aimed to determine methods used by trialists for collecting PRO data alongside HSD.

**Results:**

Of the 84 eligible studies, 52 (62%) planned to use HSD and 79 (94%) planned to collect PROs. The number of RCTs planning to use HSD for at least one outcome was 28 (54%) with 24 of these planning to use HSD as the sole data source for at least one outcome.

The number of studies planning to use HSD for primary and secondary outcomes was 10 (20%) and 21 (40%) respectively. The sources of HSD were National Health Service (NHS) Digital (*n* = 37, 79%), patient registries (*n* = 7, 29%), primary care (*n* = 5, 21%), The Office for National Statistics (ONS) (*n* = 3, 13%) and other (*n* = 2, 8%).

PROs were collected for 92% of the trials planning to use HSD. Methods for collection of PROs included in-person (*n* = 26, 54%), online (*n* = 22, 46%), postal (*n* = 18, 38%), phone (*n* = 14, 29%) and app (*n* = 2, 4%).

**Conclusions:**

HSD is being used in around two thirds of the studies but cannot yet be used to support PRO data collection within the cohort we examined. Comparison with an earlier cohort demonstrates an increase in the number of RCTs planning to use HSD.

**Supplementary Information:**

The online version contains supplementary material available at 10.1186/s13063-023-07846-4.

## Background

Randomised controlled trials (RCTs) are the gold standard for evaluating healthcare interventions [1]. RCTs usually require a lot of personnel, bespoke data collection and lengthy follow-up, thus resulting in high costs. In 2017 [2], the price of an RCT in the USA was between $40,000 and $100,000 per patient recruited.

The traditional methods of collecting data for RCTs typically involve requesting patients to provide information about their treatment by going to the trial-specific medical site and undergoing medical assessments or tests and through self-reported questionnaires, as necessary, according to the trial design, at predetermined time-points.

The use of health data collected as part of routine care, instead of, or in combination with bespoke trial data collection, may reduce the burden on participants, both patients and site staff, with an associated reduction in cost. Healthcare systems data (HSD) refers to medical information collected without having a specific research question formulated in advance. Such data can be gathered from different sources, including National Health Services (NHS) Digital, the Office for National Statistics (ONS) and disease-specific patient registries. These databases contain a large amount of information, for example, the NHS which holds comprehensive medical records for more than 65 million people that contain data recorded over 10 years. Given the resources required to undertake participant follow-up and collect bespoke clinical trial data, the efficiency that may be gained with HSD is of heightened interest.

The use of HSD in research is increasing [3] and its benefits and limitations in RCTs are being explored worldwide [4–6]. It has been argued that many common RCT limitations can be resolved by using healthcare systems data, including recruitment challenges, randomised allocation to interventions and missing data due to loss to follow-up of participants [4].

Only 3% of all UK RCTs were estimated to have successfully accessed HSD from UK-based registries between 2013 and 2018 [7]. Over half of the studies accessed this data (91/160) within the final 2 years of the cohort (2017–2018), demonstrating increasing trends in demand and availability of HSD. In 2019, a cohort of 216 ongoing trials funded by the National Institute for Health and Care Research (NIHR) were examined for their use of HSD [8]. Nearly half (47% 102/216) planned to use healthcare systems data, of which 46 (45%) aimed to use HSD as the sole source of data for one or more outcomes.

The importance of patient-reported outcome (PRO) data has been recognised [9]. However, it is as yet unknown the extent to which PRO data can be obtained from HSD, and if not, how trialists plan to collect and integrate the two sources of information. Two organisations, MRC-NIHR TMRP (https://www.methodologyhubs.mrc.ac.uk/about/tmrp/) and HDR UK (https://www.hdruk.ac.uk/), recently hosted a workshop on “What do we need to do to make Patient-Reported Outcomes (PROs) part of routinely collected health data?” [10]. Speakers at the workshop presented current research related to PRO data collection, including technical issues encountered, and patient and healthcare professional engagement, and highlighted, through open discussions, the need to embed PROs into healthcare systems data, as well as the associated opportunities and challenges.

Given the continuing focus and advances in accessing and utilising HSD, the aim of this study was to ascertain current practice amongst a United Kingdom (UK) cohort of recently funded and ongoing RCTs in relation to sources and use of healthcare systems outcome and PRO data.

## Methods

A similar study was previously undertaken which identified NIHR HTA-funded investigator-led studies in progress in 2019. We aimed to reexamine this cohort and establish a new cohort of ongoing studies added to the Journals Library after October 25, 2019. The NIHR HTA programme was selected as a major source of publicly funded clinical trials within the UK due to its use within the previous cohort for comparison. The search of the NIHR library was undertaken on June 6, 2022; search criteria are shown in Additional file 1.

NIHR HTA-funded randomised trials were eligible for the cohort if they were in progress, were described as primary research and provided access to an available protocol. Where multiple versions of the protocol were available, only the most recently published version was considered.

The following data items were extracted from all available protocols:

1. Type of trial to be conducted (randomised controlled trial, feasibility study, etc.)
2. Whether the trial involved the use of any HSD
3. The source of the HSD, where relevant
4. Whether there were PROs collected in the trial, and if so, the means of recording the PRO data.

A trial was classified as planning to use HSD if the protocol mentioned a link with any healthcare systems for any purpose. These excluded trials asking for participant consent to use this data for the purpose of future studies that are subject to further funding which has not yet been awarded. The categories for analysis were based on those used by McKay et al. [8], with amendments made as necessary (see Additional file 2).

A trial was classified as planning to use HSD as the sole data source for at least one outcome of interest if it was mentioned that data for any of the primary or secondary outcomes would be accessed using a healthcare systems data source only. Trials that aimed to use healthcare systems data to validate the results collected using bespoke data collection were not included in this category.

The use of PROs and the data collection method were recorded for each trial. The following categories were used: in-person, postal, by telephone, via text message, video conferencing, web-based and app collection. Based on their planned use, the collection methods were further categorised as either primary or secondary (for back-up reasons, e.g. if a participant did not return their postal questionnaires, members of the team would contact them by telephone). Any study within a trial (SWAT), feasibility assessment or internal pilot that related to the collection of PROs was noted. Additionally, the protocols identified in McKay’s study were reviewed to extract PRO use, not previously undertaken [8].

During the process of extracting PRO data from the protocols, both PROs and proxy-reported outcomes (i.e. those recorded by a non-medical representative on behalf of the patient) were considered, as several trials included patients who were not capable of completing outcomes on their own. However, outcomes reported by medical professionals, including nurses and professional caregivers, were excluded as they represent a professional rather than a patient-centred interpretation of the results.

## Results

**Fig. 1:**
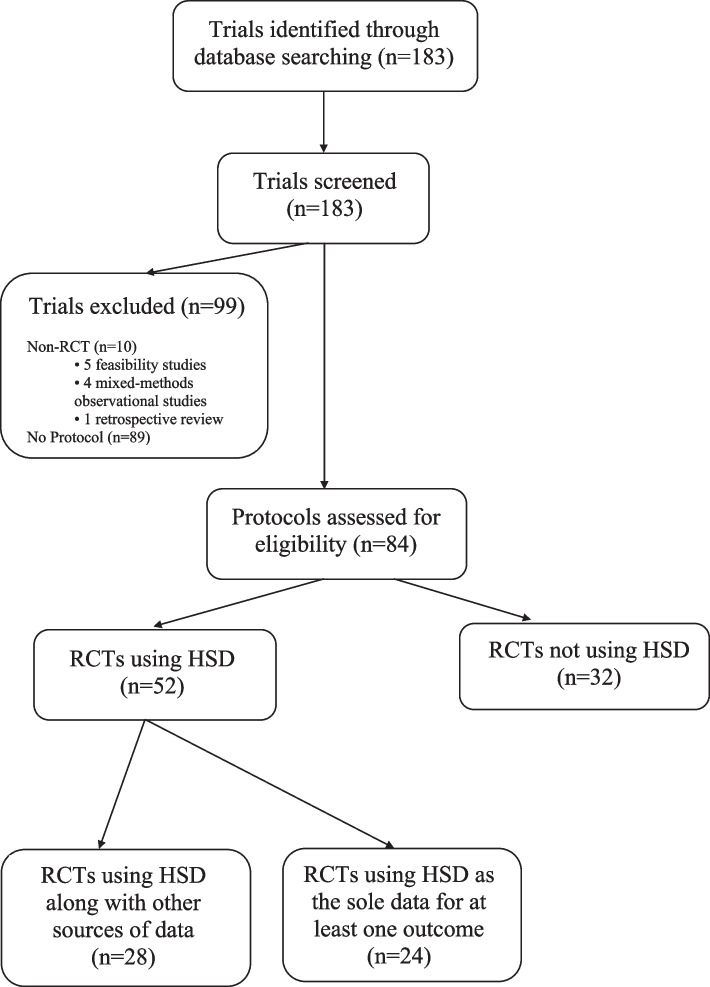
PRISMA flow diagram

There were 183 trials identified as being in progress at the time of the search (Fig. 1). Of these, 89 (48%) had no protocols and were therefore excluded. An additional 10 (5%) were not RCTs, leaving 84 (46%) protocols to be reviewed.

Fifty-two (62%) of the 84 protocols reviewed detailed plans to use healthcare systems data. Of these, 24 trials (46%) described aiming to use HSD as the sole source for at least one outcome of interest (Table 1).

**Table 1:** Overall results

	Up to 2019 [8]	2019–2022
Total number of protocols assessed for eligibility	216	84
Number of trials planning to use HSD	102 (47%^a^)	52 (62%^a^)
Number of trials planning to use HSD as the sole data for at least one outcome	46 (45%^b^)	24 (46%^b^)

^a^Percentage calculated relative to the total number of protocols seen

^b^Percentage calculated relative to the number of trials planning to use HSD

There has been an increase in the proportion of trials planning to use healthcare systems data since the original review, while the percentage of trials planning to use HSD as the only source of data for at least one outcome remains relatively similar (Table 1). There are three protocols that mention using only HSD and PROs, without any bespoke clinical data collection (Table 2).

**Table 2:** Reasons for sourcing HSD

HSD use	Up to 2019 [8]: *n* (% of 102 trials)	2019–2022: *n* (% of 52 trials)
1. Participant recruitment	N/A	4 (8%)
2. Collection of baseline data	1 (1%)	8 (15%)
3. Primary outcome (PO)^c^		
3.1 PO ascertained solely from HSD	23 (22%)	6 (12%)
3.2 PO partially ascertained from HSD	N/A	4 (8%)
4. Secondary outcomes (SOs)^c^		
4.1 SO(s) ascertained solely from HSD	35 (34%)	20 (38%)
4.2 SO(s) partially ascertained from HSD	N/A	1 (2%)
5. The use of HSD collected post-withdrawal		
5.1 All outcome data can be collected from HSD	1 (1%)	9 (17%)
5.2 Partial outcome data can be collected from HSD	17 (17%)	23 (44%)
6. For the feasibility study	12 (12%)	2 (4%)
7. Full trial data^a^ to be accessed from registries	3 (3%)	2 (4%)
8. Long-term follow-up (already budgeted in the current trial)	4 (4%)	23 (44%)
9. Health economic (HE) analysis		
9.1 HE analysis uses HSD only	4 (4%)	5 (10%)
9.2 HE analysis uses HSD alongside other sources	7 (7%)	11 (21%)
10. To be used if needed	N/A	6 (12%)
11. Other^b^	N/A	3 (6%)

*N/A* not available

^a^Full trial data includes all outcome data, along with any additional information about the patients

^b^One trial is planning to use HSD to facilitate communications; the second and third trials are planning to use HSD for partial validation of bespoke data

^c^It is unknown if McKay et al. [8] differentiated between the partial/full collection of outcomes in the cohort reviewed

Table 3 defines the sources when outcome data are obtained solely from HSD, demonstrating that many of the RCTs use multiple sources of HSD. In the current cohort of trials, 46% of the trials planning to use HSD solely for at least one outcome plan to use more than one source of healthcare systems data, while in McKay et al. [8], this percentage is 61%. The main source of HSD in both cohorts is NHS Digital; indeed, there is an increase in the proportion of trials planning to use data from NHS Digital since the original review, alongside a decrease in the use of sources like ONS and registries.

**Table 3:** HSD source for RCTs planning to use healthcare systems data as the sole data source for at least one outcome

Source	Number of trials (% of *n* trials planning to use HSD as sole data source for at least one outcome)	
	Up to 2019 [8] *(n*=46)	2019–2022 (*n*=24)
Primary care data (all regional equivalents)	8 (17%)	5 (21%)
NHS Digital^a^ (including HES and all regional equivalents)	27 (59%)	19 (79%)
ONS (and/or regional equivalents)	27 (59%)	3 (13%)
Data collected specifically for patient group or healthcare intervention (to include patient registries, mortality records, etc.)	26 (57%)	7 (29%)
Other	5 (11%)	2 (8%)

^a^Now NHS England

Table 4 illustrates the most common outcomes that were collected fully from HSD (in the current cohort only). Other outcomes mentioned include treatment failure, specific events (e.g. asthma attacks) and specific drug measurements (e.g. cumulative dose of treatment).

**Table 4:** Outcomes collected from HSD

Outcomes collected from HSD	No. of trials (% out of 24)
Mortality-related outcome	19 (79%)
Hospital admission	13 (54%)
Adverse effects	7 (29%)
Cost-related outcome	6 (25%)
Duration of hospital stay	4 (17%)
New diagnostics of interest (e.g. new cancer diagnosis)	4 (17%)
Organ support needed	3 (13%)

Table 5 describes the proportion of trials planning to collect PROs, which is similar across the two cohorts regardless of whether HSD is also used. The primary method of collection remains in-person, while postal questionnaire use has decreased. The use of online data collection has increased over time for both web-based and app approaches.

**Table 5:** Patient-reported outcomes and data collection methods

	Up to 2019 [8]	2019–2022
Collecting PROs	204/216 (94%)	79/84 (94%)
PROs and HSD	100/102 (98%)^a^	48/52 (92%)^a^
Recording of PROs		
Primary collection method^b^		
In-person	49 (49%)	26 (54%)
Phone	24 (24%)	14 (29%)
Text	2 (2%)	2 (4%)
Video	2 (2%)	3 (6%)
Post	51 (51%)	18 (38%)
Web-based (Online)	22 (22%)	22 (46%)
App	1 (1%)	2 (4%)
Number of primary methods of PRO collection used^b^		
1	48 (48%)	23 (48%)
2	34 (34%)	14 (29%)
3	9 (9%)	9 (19%)
4	2 (2%)	1 (2%)
5	0 (0%)	1 (2%)

^a^Percentage of the total number of trials planning to use HSD

^b^Percentage of the total number of trials planning to use both HSD and collect PROs

In 23% of the trials collecting both PRO data and HSD, a sub-study using PROs has been included (Table 6). Predominantly, this study assesses the PRO response rate, but the adherence to treatment and patient-reported treatment success are also examined. There were no sub-studies looking at PRO data from HSD.

**Table 6:** Sub-studies

Sub-study question	Number of sub-studies
Questionnaire response rate	
Only checking the response rate	2 (18%)
Including a “Thank you” note	2 (18%)
Including a pen	1 (9%)
Including an animated participant video	1 (9%)
Changing the questionnaire frequency	1 (9%)
Including a social retention cover letter	1 (9%)
Adherence to treatment and ACT reporting	2 (18%)
Patient-reported success rate of treatment	1 (9%)

## Discussion

The current research has three key findings, based on the aim of comparing the current trials in progress and the ones identified in McKay et al. [8]. First, there has been an increase in the number of trials planning to use HSD for any reason, from 47% in trials ongoing in 2019 [8] to 62% in trials started between 2019 and 2022. Second, survival and hospital admission were the outcomes most commonly to be collected from HSD alone.

Finally, PROs are measured in nearly all trials, but, within the current cohort, none are collecting PRO data from HSD. The importance of integrating PROs within HSD was recently discussed at the TMRP-HDRUK North workshop [10]. While there is a need to further explore the topic, the online collection of PRO data could be potentially integrated into HSD databases, such as patient registries. Currently, it can be observed that the preference for an online collection method has increased.

There are several strengths and limitations in the current research. The source of the trials and the inclusion/exclusion criteria match the previous study [8] facilitating comparison. However, all the trials included are NIHR funded, which might not completely be representative of all the RCTs currently in progress in the UK, or beyond.

Data up-cycling refers to reusing information already collected. As more trialists begin to access HSD, the amount of data available for research is becoming more widely recognised. There are potential issues to be considered when using healthcare systems data. The recently published COMORANT-UK study [11] has released a prioritised list of challenges to be addressed regarding HSD. The domains of the questions included data access, data collection and outcome selection.

Several recent publications [12, 13] have highlighted issues regarding access to data. Powell et al. [13] described trying to access 14 databases in order to gather information about 98 participants. The results suggested that secondary care data, although challenging in terms of application process, was available to access, whereas primary care data had limited accessibility and non-clinical datasets were not accessible. An update to this review is currently underway [14], aiming to further evaluate the degree of agreement between bespoke and HSD in recent UK clinical trials.

HSD related to adverse effects is being collected in almost a third of trials. Another key point previously discussed [7, 12, 13] is the timeliness of data. Data collected from healthcare systems usually involves a delay between the recording of the data and it being supplied to the trial team; for example, Hospital Episode Statistics (HES) data take approximately 3 months to be provided [12].

The PRIMORANT study sought to address two of the prioritised questions from the COMORANT study: “How should the trials community decide when routinely collected data for outcomes is of sufficient quality and utility to replace bespoke data collection?” and “What are the best methods to communicate and build trust with trial participants (and the public) about how their routinely collected data will be used?”. While the second part was approached through exploring different methods of communicating to the public, the work around the first question resulted in a list of issues to consider (under review). This list explored the necessary changes to the trial structure and highlighted aspects that should be considered before deciding to use HSD. These include terminology, feasibility, internal pilot, onward data sharing and data archiving. Following the publication of the PRIMORANT paper, it will be of interest to explore any resulting changes in the extent and nature of HSD use in trials.

## Conclusion

Our research examined a cohort of ongoing RCTs and described their planned use of healthcare systems data and patient-reported outcomes. The proportion of RCTs accessing HSD has increased over time, although the proportion of planning to use it as the sole source of data for at least one outcome of interest has remained similar. This suggests the increased interest in HSD, while being aware of the current barriers of solely relying on this data. Future snapshots of HSD use in trials will be beneficial in relaying its evolution. Further research exploring the reasoning behind choosing whether to use HSD in RCTs, or not, would be useful.

The increase in online data collection for PROs supports the potential for remote data collection. This suggests it may be possible to integrate PRO with clinical data collected from HSD in a single system. Further work is needed to enable this integration, with the benefit of reducing the burden of research participation.

### Supplementary Information

**Additional file 1.** Search Criteria. Presents the search criteria used in the NIHR Journals Library.

**Additional file 2.** Changes in categories for the data collected in 2022 compared to the previous review in 2019. Presents the changes made in categories for the data collected in 2022 compared to the previous review.

## Data Availability

The datasets generated and/or analysed during the current study are available in the “The use of healthcare systems data for RCTs - data” folder, [10.6084/m9.figshare.24158535].

## Abbreviations

HE: Health economic
HES: Hospital Episode Statistics
HSD: Healthcare Systems Data
NHS: National Health Service
NIHR: National Institute for Health and Care Research
ONS: Office for National Statistics
PO: Primary outcome
PRO: Patient-reported outcome
RCT: Randomised controlled trial
SO: Secondary outcome
SWAT: Study within a trial
