# Supplementary material for: The use of healthcare systems data for RCTs
Source: Trials. 2024 Jan 29;25:95. doi: 10.1186/s13063-023-07846-4 (PMC10826061; doi:10.1186/s13063-023-07846-4)
Supplement: Supplementary file 1 — Additional file 1. Search Criteria. Presents the search criteria used in the NIHR Journals Library. [file 13063_2023_7846_MOESM1_ESM.docx]

Additional file 1 **Search criteria**

**Search criteria**

The following search criteria was used on the NIHR Journals Library:

1. “Search term/Keywords”: Random

2. “Research Type”: Primary Research

3. “Programme”: Health Technology Assessment

4. “Project Type”: Research in progress and Waiting to start *

*The final search criterion (number 4) was applied after the initial search was processed as it was available as a filter on the results page.

Due to changes in the NIHR website, in order to filter the project type, there need to be changes to the website link, as their current filters no longer allow for this selection. For the trials that are currently in progress, the following code should be added at the end of the link:

“&selected_facets=status:%22Research%20in%20progress%22”

For the trials that are waiting to start, the following code should be added at the end of the link:

“&selected_facets=status:%22Waiting%20to%20start%22”
