# Supplementary material for: The use of healthcare systems data for RCTs
Source: Trials. 2024 Jan 29;25:95. doi: 10.1186/s13063-023-07846-4 (PMC10826061; doi:10.1186/s13063-023-07846-4)
Supplement: Supplementary file 2 — Additional file 2. Changes in categories for the data collected in 2022 compared to the previous review in 2019. Presents the changes made in categories for the data collected in 2022 compared to the previous review. [file 13063_2023_7846_MOESM2_ESM.docx]

Additional file 2 **Changes in categories for the data collected in 2022 compared to the previous review in 2019**

Categories added:

1. the recording of HSD use for patient recruitment.
2. the division between full and partial collection of both primary and secondary outcomes.

Categories combined:

- 1. 2019 categories 'Registry trial: As the sole source of outcome data with purpose-built Module to collect remaining outcome data', 'Registry trial: All outcome data collected through multiple RCHD sources except for questionnaire data' and 'Registry trial: All outcome data collected through multiple RCHD sources except for some baseline data, questionnaire data and other patient-reported data' were combined into ‘Full trial data to be accessed from registries’ in 2022.
  2. 2019 categories 'Supplementing data collection for withdrawn patients (consent asked for at time of withdrawal)’, 'Supplementing data collection for lost-to-follow-up patients' and 'Supplementing data collection for withdrawn patients (consent NOT ASKED FOR at time of withdrawal)' were combined into ‘The use of HSD collected post withdrawal, Partial outcome data can be collected from HSD’ in 2022.
